# Blu-Ray-Based Quantification of CD98+ Extracellular Vesicles for Early Detection of Hepatocellular Carcinoma

**DOI:** 10.3390/cancers18071086

**Published:** 2026-03-26

**Authors:** Su-Liang Chen, Yong Seng Low, Bo-Ru Huang, Che-Hao Lu, Wei-Chun Lan, Ren-Huang Wu, Hsing-Ying Lin, Andrew Yueh, En-Chi Hsu

**Affiliations:** 1 Institute of Molecular and Genomic Medicine, National Health Research Institutes, Miaoli County 350, Taiwan; gloria@nhri.edu.tw (S.-L.C.); yongseng31@nhri.edu.tw (Y.S.L.); sta780604@nhri.edu.tw (B.-R.H.); 2 Institute of Biomedical Engineering, National Tsing-Hua University, Hsinchu City 300, Taiwan; luchehao@gmail.com (C.-H.L.); s221038388@gmail.com (W.-C.L.); hy.lin@mx.nthu.edu.tw (H.-Y.L.); 3 Institute of Biotechnology and Pharmaceutical Research, National Health Research Institutes, Miaoli County 350, Taiwan; wu040519@nhri.edu.tw (R.-H.W.); andrewyueh@nhri.edu.tw (A.Y.)

**Keywords:** hepatocellular carcinoma, CD98, extracellular vesicles, exocounter technology, cancer early detection

## Abstract

Hepatocellular carcinoma (HCC) remains a major global health burden, with an increasing incidence driven by metabolic syndrome-related cases. Early detection is critical; however, current diagnostic tools, including ultrasonography and alpha-fetoprotein (AFP), lack adequate sensitivity and specificity, particularly for non-viral HCC. In this “discovery-stage” study, we identified CD98, a transmembrane oncoprotein, as a robust biomarker highly expressed across all HCC stages, independent of etiological factors. CD98 is enriched on extracellular vesicles (EVs) released by HCC cells and can be accurately quantified using Blu-ray-based ExoCounter technology. In plasma samples from patients with early-stage (stage I/II) non-viral HCC (*n* = 136) and healthy controls (*n* = 50), CD98+ EV levels were significantly elevated (*p* < 0.001) and demonstrated strong diagnostic performance (AUC = 0.743; sensitivity 64%, specificity 86%). Importantly, CD98+ EV levels detected small tumors (≤3 cm) with 59% sensitivity, outperforming AFP (33%). These findings highlight circulating CD98+ EVs as a promising, noninvasive biomarker for early non-viral HCC detection, providing a clinically applicable platform that integrates EV quantification with a novel anti-CD98 monoclonal antibody for precision oncology.

## Simple Summary

Early detection of liver cancer remains challenging, particularly in patients without viral hepatitis, as current screening tools have limited accuracy. In this study, we evaluated a novel blood-based biomarker, CD98, carried by extracellular vesicles released from cancer cells. We found that the levels of CD98+ extracellular vesicles were significantly increased in patients with early-stage liver cancer, demonstrating superior diagnostic performance compared to commonly used markers. These results suggest that CD98+ extracellular vesicles may serve as a valuable noninvasive biomarker to complement existing methods and improve the early identification of liver cancer in clinical practice.

## 1. Introduction

Liver cancer is the sixth most common malignancy and the third leading cause of cancer-related mortality worldwide, with hepatocellular carcinoma (HCC) accounting for approximately 85% of all cases [1]. The major etiological factors for HCC include chronic hepatitis B (HBV) and hepatitis C (HCV) infections, alcohol consumption, aflatoxin exposure, and metabolic disorders [2]. Nowadays, organized surveillance systems for viral hepatitis and successful HBV vaccination programs have substantially reduced the incidence of virus-related HCC [3]. However, the prevalence of metabolic syndrome-associated HCC arising from nonalcoholic fatty liver disease (NAFLD) and nonalcoholic steatohepatitis (NASH) continues to rise. This is particularly evident in Western populations, where the lack of a surveillance system comparable to that for viral hepatitis presents a major clinical challenge [4]. Global incidence is projected to reach 1.4 million cases by 2040 [5], establishing HCC as a growing health crisis. Notably, metabolic syndrome-related HCC often exhibits poorer survival compared to virus-related cases. Patient outcomes are closely linked to tumor stage; according to the Barcelona Clinic Liver Cancer (BCLC) staging system [6], those with very early (BCLC 0, tumor size ≤ 2 cm) or early-stage (BCLC A, tumor size ≤ 3 cm) disease have a 5-year survival rate of ~40%, compared to only 10–15% for advanced (BCLC C) and terminal stages (BCLC D). Thus, improving early detection represents the most effective strategy to enhance HCC prognosis and expand access to curative therapies, such as resection and ablation.

The current surveillance standards—abdominal ultrasonography (US) and serum alpha-fetoprotein (AFP) measurement—are limited by suboptimal sensitivity and specificity. Although US is noninvasive and cost-effective, its sensitivity for tumors smaller than 2 cm is only 63% in HBV patients and decreases to 47% in those with cirrhosis or steatohepatitis due to fibrotic distortion of liver architecture [7]. AFP, a fetal glycoprotein re-expressed in HCC, is widely used but lacks specificity, as elevated levels are also observed in other malignancies, chronic liver diseases, and pregnancy [8]. The diagnostic performance of AFP (cutoff ≥ 20 ng/mL) varies, with a sensitivity of 41–65% and a specificity of 80–94% [9]. For small tumors (≤3 cm), AFP sensitivity drops to ~25% [10]. Lowering the threshold increases false positives, whereas higher cutoffs (≥200 ng/mL) frequently fail to detect early lesions. Additional serum markers, including AFP-L3 [11], des-γ-carboxy prothrombin (DCP or PIVKA-II) [12] and glypican-3 (GPC3) [13], have shown limited clinical utility. While the combined use of AFP and PIVKA-II marginally improves detection [10,14], a highly sensitive and specific blood-based biomarker for early HCC remains an urgent unmet need.

Extracellular vesicles (EVs)—nanometer-scale, bilayered vesicles secreted by cells—have emerged as promising candidates for liquid biopsy-based diagnostics. EVs, particularly exosomes (30–150 nm), encapsulate proteins, nucleic acids, and lipids that reflect their cellular origin and remain stable in circulation. Numerous exosomal biomarkers for HCC have been reported, including microRNAs (e.g., miR-21, miR-122), long noncoding RNAs (e.g., LINC00161, SNHG1), circular RNAs (e.g., circ_0070396, circ_0028861), and proteins (e.g., G3BP, S100A4, CAP1) [15].

CD98 (SLC3A2), a single-pass transmembrane glycosylated oncoprotein, is overexpressed in various cancers—including lung, colorectal, and pancreatic malignancies—and is also highly upregulated in HCC, even at early stages. Functionally, CD98 regulates amino acid transport, integrin signaling, and tumor microenvironment remodeling by interacting with LAT1, CD147, and integrin β1, thereby activating oncogenic pathways such as FAK/PI3K/AKT, MAPK/ERK, Wnt/β-catenin, and mTOR [16]. Monoclonal anti-CD98 antibodies have demonstrated tumor-suppressive effects in HCC xenograft models [17], underscoring its importance in hepatocarcinogenesis.

Given its surface localization and abundance in extracellular vesicles, we hypothesized that CD98 could serve as a cancer-specific EV surface biomarker. Here, we demonstrate that CD98 expression is elevated at both the mRNA and protein levels across all stages of HCC. We further show that CD98 is detectable on EVs secreted from HCC cell lines and in plasma-derived EVs from patients with early non-viral HCC. Using Blu-ray-based ExoCounter technology, we established a quantitative “CD98+ EV Index” that effectively distinguishes early-stage HCC from healthy controls. Moreover, this index provided superior diagnostic performance for non-viral early-stage HCC (tumor size ≤ 3 cm). These findings suggest that circulating CD98+ EVs represent a promising, noninvasive biomarker for early HCC detection.

## 2. Materials and Methods

### 2.1. Cell Culture

Human hepatocellular carcinoma (HCC) cell lines HepG2 (BCRC RM60025), Hep3B (BCRC 60434), and PLC5 (BCRC 60223) were obtained from the Bioresource Collection and Research Center (BCRC, Food Industry Research and Development Institute, Taiwan) and authenticated by DNA fingerprinting. HepG2 and Hep3B cells were maintained in Eagle’s Minimum Essential Medium (EMEM; Thermo Fisher Scientific, Carlsbad, CA, USA), while PLC5 cells were cultured in Dulbecco’s Modified Eagle’s Medium (DMEM; Thermo Fisher Scientific). All media were supplemented with 10% (*v*/*v*) fetal bovine serum (FBS; Cytiva, Marlborough, MA, USA), 1% penicillin–streptomycin (Thermo Fisher), and 1% GlutaMAX (Thermo Fisher). Cells were cultured at 37 °C in a humidified incubator with 5% CO_2_.

### 2.2. Clinical Samples

The mRNA expression levels of CD98 in normal liver tissue (*n* = 175) versus HCC (*n* = 421) patient samples from GTEx [18] and TCGA HCC (LIHC) [19] datasets were analyzed using the TNMplotter web tool (20). mRNA expression levels of CD98 across different stages (BCLC system), recurrence, tumor size, blood AFP, and PIVKA-II values were obtained from the CLCA cohort [20] via the cBioPortal platform [21]. The liver hepatocellular carcinoma tissue microarray (LV1501a) was purchased from TissueArray.com (Ijamsville, MD, USA) and included TNM classification and pathological staging. The array comprised 61 HCC and 5 normal liver tissues with duplicate cores per case.

Plasma samples were collected from HBV/HCV-negative patients with stage I/II HCC (*n* = 136) and healthy donors (*n* = 50) through the National Biobank Consortium of Taiwan (NBCT). Only patients with histologically confirmed stage I/II HCC (T1N0M0/T2N0M0) were included, while those with stage III or higher disease or virus-related HCC were excluded. The study was approved by the Ethics Committee of the National Health Research Institutes (approval no. EC1120205-E). Plasma samples were stored at −80 °C, thawed on ice, and centrifuged at 3000× *g* for 15 min to remove cells, platelets, and cellular debris. The supernatant was then centrifuged at ~13,600× *g* for 10 min prior to exosome isolation.

### 2.3. Development and Production of CD98 Rabbit Monoclonal Antibody (NHRI#20D5)

The extracellular domain (ECD) of human CD98 with a His-tag was used for rabbit immunization. Single-B-cell screening was performed via CD98 ELISA (~3000 monoclonal antibodies generated), followed by evaluation of the top 93 positive clones through iterative positive selection (CD98-coated plates) and negative selection (6xHis-tag irrelevant protein-coated plates) at GenScript Biotech Corporation (Piscataway, NJ, USA). The optimal clone, NHRI#20D5, was selected based on its specific binding to the CD98 ECD without cross-reactivity to the His-tag. mRNA from NHRI#20D5-secreting B cells was reverse-transcribed, and V_H_/V_L_ regions were amplified by nested PCR to construct full-length antibody expression plasmids in a pcDNA3.4 backbone. Expi-293F™ cells (A14528, Thermo Fisher) were transfected with heavy- and light-chain plasmids (1:1 ratio) using FectoPRO^®^ (Polyplus, Illkirch, France). After 6 days, antibodies were purified from culture supernatants using protein A Sepharose chromatography (GenScript). Eluted antibodies were neutralized (0.1 M sodium citrate, pH 3.0; Tris-HCl, pH 9.0), concentrated using Microsep Advance centrifugal filters (Pall, Port Washington, NY, USA), and assessed for purity by 12% SDS–PAGE and BCA assay (Thermo Fisher).

### 2.4. Immunohistochemistry (IHC)

Slides were deparaffinized, rehydrated through graded ethanol, and subjected to antigen retrieval in sodium citrate buffer (10 mM, pH 6.0). Endogenous peroxidase activity was quenched with 3% H_2_O_2_, and nonspecific binding was blocked with 2.5% goat serum (MP-7451, Vector Labs, Newark, CA, USA) for 1 h. Slides were incubated overnight at 4 °C with rabbit anti-CD98 antibody (1:100, ab307587, Abcam, Cambridge, UK), followed by HRP-conjugated anti-rabbit secondary antibody (MP-7451, Vector Labs) for 1 h at room temperature. Signals were visualized using DAB substrate (1:50, SK-4105, Vector Labs), and sections were counterstained with hematoxylin, dehydrated, and mounted for digital scanning.

### 2.5. Western Blot

Whole-cell lysates and EV fractions were prepared with RIPA buffer (Visual Protein, Taipei City, Taiwan) supplemented with protease/phosphatase inhibitors (Thermo Fisher). Protein concentrations were determined using a BCA kit (Thermo Fisher). Exosomes from HepG2, Hep3B, and PLC5 cells were isolated using ExoQuick™ (System Biosciences, Palo Alto, CA, USA) and resuspended in PBS. Samples were mixed with 4× loading buffer, heated at 95 °C for 5 min, and resolved on 4–12% SDS–PAGE (Thermo Fisher). Proteins were transferred to NC membranes (Cytiva), blocked with 5% skim milk (BD) for 1 h, and incubated overnight at 4 °C with primary antibodies, which were used at a 1:1000 dilution: CD9 (GeneTex, Irvine, CA, USA), CD63 (ABclonal, Woburn, MA, USA), CD81 (GeneTex), Flotillin1 (Cell Signaling, Danvers, MA, USA), TSG101 (Cell Signaling), ApoA1 (Cell Signaling), Calnexin (GeneTex), β-Actin (Santa Cruz, Dallas, TX, USA), GAPDH (Santa Cruz), and CD98 (abcam, Cambridge, UK). HRP-conjugated secondary antibodies (Thermo Fisher) were applied for 1 h, and signals were developed with SuperSignal™ West Pico PLUS or Atto (Thermo Fisher) and visualized using a ChemiDoc MP system (Bio-Rad, Hercules, CA, USA).

### 2.6. Flow Cytometry

HCC cells (1 × 10^6^) were washed, centrifuged, and incubated with anti-CD98 antibodies (Abcam ab307587; Novus NBP2-36491SS; NHRI#20D5) or rabbit IgG control (bs-0295P, Bioss Antibodies, MA, USA) for 1 h on ice, followed by Alexa Fluor 488-conjugated goat anti-rabbit IgG (111-545-003, Jackson ImmunoResearch, West Grove, ME, USA) for 1 h at 4 °C. Cells were washed, resuspended in PBS with 7-AAD (559925, BD Biosciences, San Jose, CA, USA) for 10 min, filtered (70 µm mesh), and analyzed on an Attune NxT flow cytometer (Thermo Fisher). Viable single cells were gated, and data were analyzed with FlowJo v10 (FlowJo LLC, Ashland, OR, USA). For antigen neutralization, anti-CD98 antibodies (0.5 µg NHRI#20D5) were incubated with or without recombinant CD98 protein (5 µg CD98 extracellular domain in 100 µL PBS) at 4 °C for 60 min. After incubation, both neutralized and unneutralized antibodies (0.5 µg) were incubated with HepG2 cells in 100 µL PBS at 4 °C for 60 min. Subsequent procedures followed the same protocol as described in the Flow Cytometry Analysis Section, including incubation with the secondary antibody for 60 min at 4 °C, 7-AAD staining for viability, filtration through a 70-µm mesh, and flow cytometric analysis.

### 2.7. Cell Culture Media EV Isolation by ExoQuick-TC

EVs were isolated from conditioned media of HepG2, Hep3B, and PLC5 cells. Cells were seeded in 10 cm dishes (4 × 10^6^ HepG2; 1.2 × 10^6^ Hep3B; 2 × 10^6^ PLC5 cells) and cultured with 5% exosome-depleted FDhPL (AventaCell BioMedical, Kent, WA, USA). After 48 h, conditioned media were collected, centrifuged (2000× *g*, 15 min, 4 °C), filtered (0.22 µm), concentrated (6-fold, 100 kDa MWCO, Cytiva), and precipitated with ExoQuick-TC (System Biosciences) per the manufacturer’s protocol.

### 2.8. Plasma EV Isolation by SmartSEC HT Kit and Transmission Electron Microscopy (TEM) Imaging

Plasma samples (250 µL) were first thawed and subjected to low-speed centrifugation (3000× *g*, 15 min). The supernatant was then further centrifuged at ~13,600× *g* for 10 min prior to extracellular vesicle (EV) isolation using the SmartSEC HT kit (System Biosciences) according to the manufacturer’s protocol. Filtered HEPES buffer (20 mM, pH 7.4) was mixed 1:1 with plasma, incubated for 30 min at room temperature, and centrifuged (500× *g*, 2 min) to collect fractions. All centrifugation treatments were performed using an Eppendorf 5910 Ri centrifuge with a swing-bucket rotor. Fractions were stored at −80 °C until analysis.

To validate the EV morphology after purification, parts of plasma EVs were adsorbed onto a glow-discharged carbon-coated copper grid, washed, and negatively stained with 1% phosphotungstic acid (PTA). The grids were then examined using a transmission electron microscope, FEI Tecnai G2 F20 S-TWIN (FEI Company, Hillsboro, OR, USA), operated at an accelerating voltage of 120 kV.

### 2.9. EV Quantification by Nanoparticle Tracking Analysis (NTA)

NTA was conducted using a ZetaView x30 instrument (Particle Metrix, Inning am Ammersee, Germany) calibrated with polystyrene standards. Samples were diluted in PBS to 50–300 particles/frame, illuminated with a 640 nm laser, and recorded at 11 positions (1 s each). Particle size and concentration were analyzed using ZetaView software (v8.05.16 SP7). Instrument parameters were held constant (autofocus, camera sensitivity 70, shutter 100, 22 °C).

### 2.10. CD98+ EV Quantification by ExoCounter

EV detection was performed using a 16-well optical disc coated with the in-house anti-CD98 antibody (NHRI#20D5). Each reaction contained 12.5 µL of sample mixed with 37.5 µL PBS and incubated for 2 h at 37 °C. Wells were washed with PBST, followed by addition of anti-CD9 and anti-CD63 beads (CosmoBio, Tokyo, Japan) under a magnetic field. After washing and drying, discs were analyzed on the ExoCounter system (JVCKENWOOD Corporation, Yokohama, Japan). Positive control: Novus NBP2-49854 (1 µL in 50 µL PBS); negative control: PBS. The CD98+ EV score was normalized to the negative control from each disc.

### 2.11. Single-EV Optical Imaging and Confocal and STED Imaging

Anti-CD9, anti-CD63, and anti-CD98 (NHRI#20D5) antibodies were conjugated to Alexa Fluor 350, 555, and 647 dyes (A20180, A20186, A20187, NovaFluor™ Antibody Conjugation Kits, Invitrogen, Waltham, MA, USA) following the manufacturer’s instructions. Total HepG2-derived EVs were labeled with Alexa Fluor 488 TFP ester (A37570, Invitrogen). Stained EVs were purified using Zeba Micro Spin columns (40 kDa MWCO, Cytiva) and qEV 70 nm columns (Izon Science, Christchurch, New Zealand). Fluorescently labeled EVs were immobilized on PTFE-printed glass slides (Electron Microscopy Sciences, Hatfield, PA, USA) and imaged with an Olympus IX83 fluorescence microscope (Olympus, Tokyo, Japan). Images were captured sequentially under FITC (TFP), DAPI (CD9), TRITC (CD63), and Cy5 (CD98) filter sets. For image analysis, multichannel fluorescence images (20×, *n* = 3) were processed using custom Python scripts (Python 3.11.9). EVs were segmented in the FITC channel (global triangle threshold, morphological filtering, area gating), and the derived mask was applied to all other channels. For each field of view (FOV), total EV count, CD9+/CD63+/CD98+ counts, and co-expression frequencies were extracted after local background subtraction. Parameters were held constant across experiments for reproducibility. To validate the EV quality after purification, parts of the HepG2-derived EVs were stained with the PKH-26 lipophilic dye following the manufacturer’s instructions. To remove unbound dye, the stained EV suspension was washed and concentrated by an Amicon Ultra-4 centrifugal filter tube to a density suitable for single-vesicle imaging. Fluorescence imaging was performed using a Leica SP8 gSTED microscope equipped with a 100× objective lens, and super-resolution imaging was achieved using a 660 nm depletion laser.

### 2.12. Statistical Analyses

Recurrence-free survival (RFS) was analyzed using Kaplan–Meier curves and log-rank tests. Continuous data are presented as mean ± SD; categorical data as frequency and percentage. Intergroup comparisons were performed using unpaired two-tailed Student’s *t* tests with Welch’s correction or Chi-square tests, as appropriate. Statistical analyses and graphs were generated using GraphPad Prism 9 and DATAtab web-based statistics calculator. *p* ≤ 0.05 was considered statistically significant. Receiver operating characteristic (ROC) analysis was performed using SRplot [22]. The cut-offs are calculated based on Youden index, defined as the point maximizing sensitivity + specificity − 1, to maximize both sensitivity and specificity. Z-score tests were used to compare two population proportions, and 5-fold stratified cross-validation was used to evaluate the performance of our model.

## 3. Results

### 3.1. CD98 Is Highly Expressed Across All Stages of HCC

**Figure 1:**
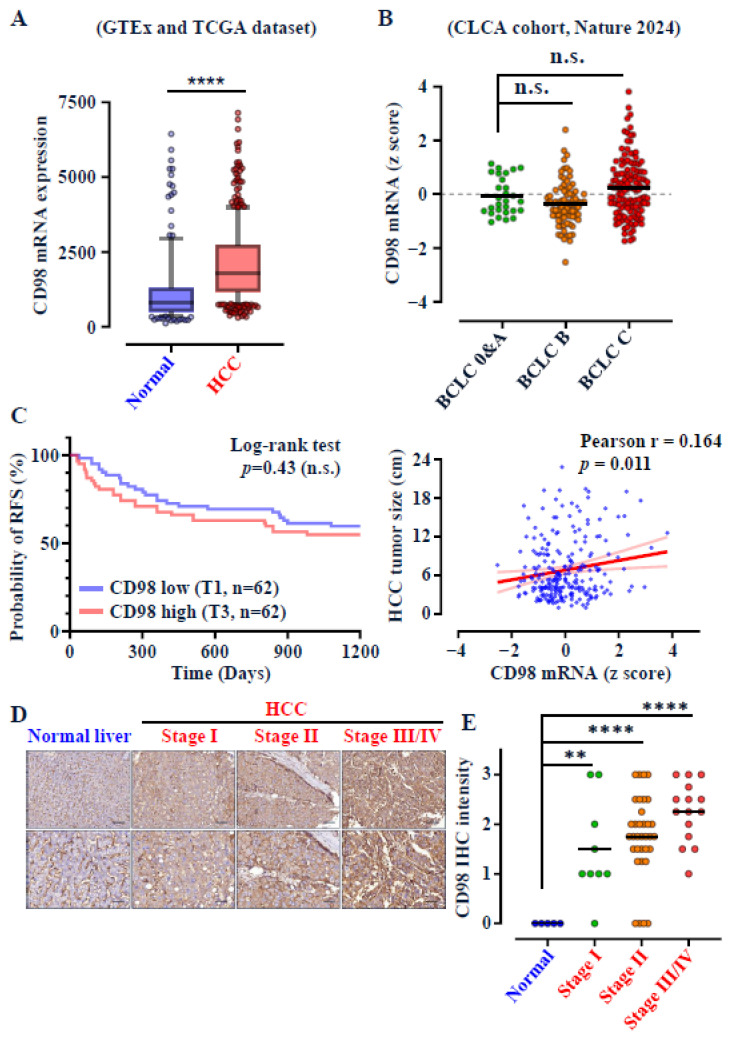
CD98 is highly expressed across all stages of HCC and is independent of survival and tumor size. (**A**) mRNA expression levels of CD98 in normal liver tissue (*n* = 175) versus HCC (*n* = 421) patient samples from GTEx and TCGA datasets are shown as box plots. (**B**) From the CLCA cohort [20] obtained via the cBioPortal platform [21], CD98 mRNA expression in HCC tumors at early stages (BCLC 0 & A, *n* = 28) versus late stages (BCLC B, *n* = 81; BCLC C, *n* = 130) is shown as dot plots. Recurrence-free survival (RFS) of HCC patients with lower CD98 mRNA levels (T1 subgroup, *n* = 62) compared with those with higher CD98 levels (T3 subgroup, *n* = 62) was analyzed by log-rank test. (**C**) Scatter plot of CD98 mRNA z-scores showed no significant Pearson correlation with tumor size (cm). (**D**) Representative images of CD98 IHC in HCC TMA (LV1501a) across stages are shown. Scale bars = 100 µm and 50 µm, respectively. IHC staining intensity for CD98 was scored as 0 (negative), 1 (low), 2 (medium), or 3 (high) and plotted. (**E**) Dot plot of CD98 IHC scores. n.s. = not significant, ** = *p* < 0.01, **** = *p* < 0.0001 (Student’s *t* test).

**Figure 2:**
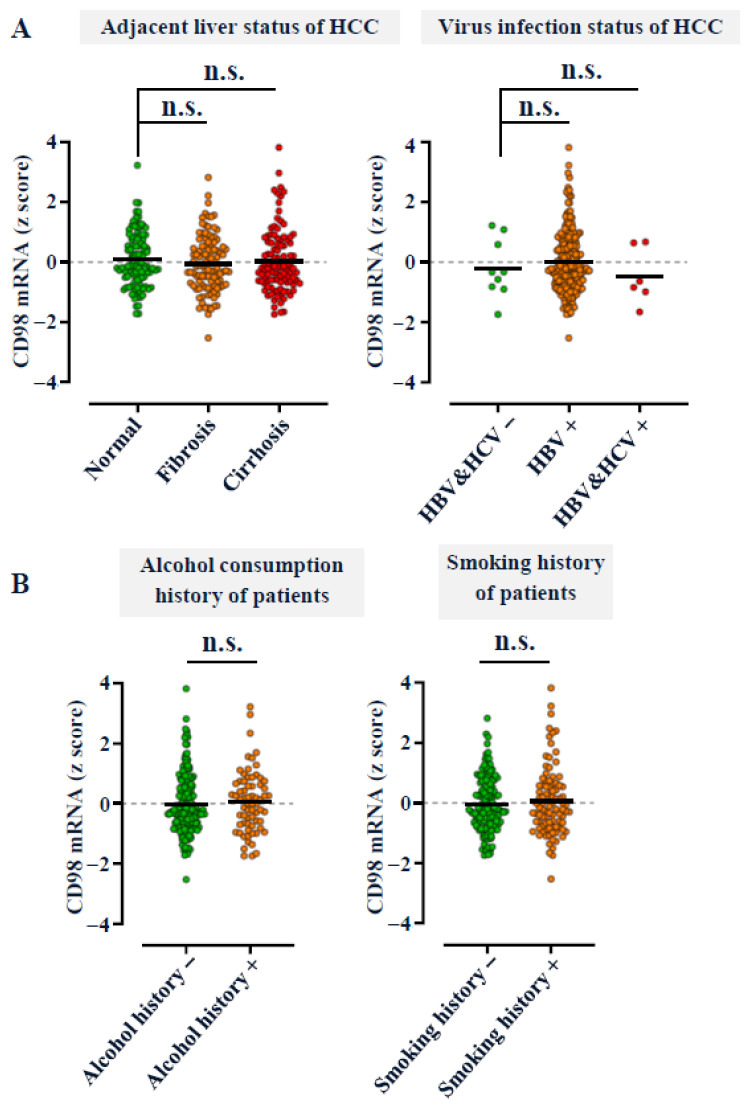
CD98 expression is independent of current etiological factors associated with HCC development. (**A**,**B**) From the CLCA cohort [20] obtained via the cBioPortal platform [21], CD98 mRNA expression levels in HCC with diverse etiological factors, including fibrosis, cirrhosis, viral infection, alcohol consumption, and smoking history, were compared. Data are shown as dot plots. No statistical significance (n.s.) was observed between groups (Student’s *t* test).

To evaluate the expression pattern of CD98 in hepatocellular carcinoma (HCC), we first analyzed transcriptomic datasets from GTEx [18] and the public TCGA HCC (LIHC) cohort [19] using the TNMplot web tool [23]. CD98 mRNA was significantly upregulated in HCC tissues compared with normal liver (*p* < 0.0001) (Figure 1A). Consistent findings were observed in the public CLCA cohort using the Barcelona Clinic Liver Cancer (BCLC) staging system [20], where CD98 expression remained consistently high across all disease stages (BCLC 0–C) (Figure 1B). While CD98 was not predictive of overall survival or associated with tumor size (Figure 1C), its stage-independent expression pattern suggests potential as a diagnostic, rather than prognostic, biomarker for early HCC. Immunohistochemical (IHC) analysis of commercial tissue microarrays (AJCC TNM system) further confirmed strong cytoplasmic membrane CD98 staining in tumors at all stages, in contrast to weak sinusoidal endothelial and negative hepatocytic staining in normal liver (Figure 1D). Quantitative scoring revealed significant upregulation of CD98 even in early-stage (Stage I) HCC, with only a modest increase in later stages (Figure 1E). Importantly, CD98 expression exhibited an early and independent upregulation at both the mRNA and protein levels across both BCLC and AJCC staging systems. To exclude confounding by known etiological factors, we examined CD98 levels across fibrosis, cirrhosis, viral status, alcohol use, and smoking history using the CLCA dataset [20]. CD98 expression was unaffected by these variables (Figure 2A,B), indicating its independence from diverse HCC risk factors and broad diagnostic applicability. In the same cohort, current blood biomarkers AFP and PIVKA-II showed limited sensitivity for early HCC detection (AFP: 12.8% or 29.8%; PIVKA-II: 38.5%), based on standard clinical cut-offs for AFP (20 or 200 ng/mL) [24,25] and PIVKA II (40 mAU/mL) [14] (Supplementary Figure S1), underscoring the unmet clinical need for improved early diagnostic markers.

### 3.2. CD98 Is Detected in Secreted Extracellular Vesicles from HCC Cell Culture Media and Patient Plasma

**Figure 3:**
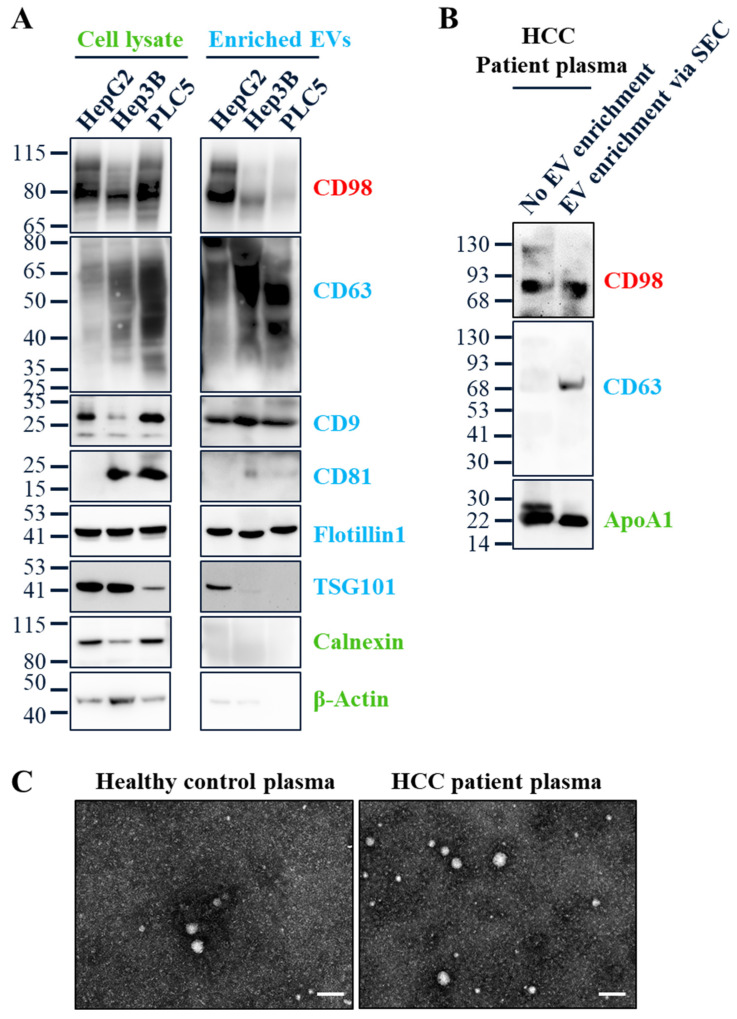
CD98 is an abundant and detectable EV surface protein in HCC cell lines and patient plasma. (**A**) Protein levels of CD98, five EV markers (CD63, CD9, CD81, Flotillin1, TSG101), and two cellular proteins (Calnexin and β-actin) in HCC cell lines or their secreted EVs from HepG2, Hep3B, and PLC5 cells via ExoQuick-TC kit were analyzed by Western blot. (**B**) Protein levels of CD98, CD63, and ApoA1 with or without EV enrichment via Size Exclusion Chromatography (SEC) in HCC patient plasma were analyzed by Western Blot. (**C**) Illustration of EV morphology of plasma after EV enrichment via SEC from the healthy control and HCC patient using transmission electron microscopy (TEM). Scale bar is 100 nm. The uncropped blots are shown in Figures S5 and S6.

CD98 has been identified as an abundant extracellular vesicle (EV) surface protein in the ExoCarta and Vesiclepedia databases [26]. Given its robust expression in HCC tissues, we next examined CD98 in EVs secreted by three HCC cell lines: HepG2 (non-viral), and Hep3B and PLC5 (HBV-integrated). EVs were enriched from culture media using the ExoQuick-TC kit. Western blotting confirmed strong CD98 expression in all cell lysates, with detectable CD98 in EV fractions, most abundantly from HepG2 cells (Figure 3A). EV identity was validated by positive markers (CD63, CD9, CD81, Flotillin-1, TSG101) and the absence of the ER protein Calnexin. Notably, we observed that CD63 and CD9 were abundant in HCC-derived EVs, whereas CD81 was not. This is consistent with clinical findings showing the loss of CD81 in poorly differentiated and metastatic HCC [27].

Enriched plasma EVs from patients were collected using SmartSEC HT and validated by Western blot. As shown in Figure 3B, CD98 was detectable in the plasma EVs of HCC patients. EV identity was confirmed by CD63 and ApoA1, a common plasma protein and EV-associated marker [28]. Furthermore, transmission electron microscopy (TEM) demonstrated that purified plasma EVs exhibited typical morphology (Figure 3C).

Since CD98 is a single-pass transmembrane protein, we optimized antibody recognition for its native extracellular domain. HepG2-derived EVs were selected for evaluation due to their high CD98 abundance and our focus on non-viral HCC. Among three tested antibodies—Abcam (ab307587), Novus (NBP2-36491SS), and NHRI#20D5 (our in-house monoclonal antibody)—flow cytometry revealed that NHRI#20D5 exhibited the highest binding affinity and specificity, which was completely blocked by pre-incubation with the recombinant CD98 ectodomain (Supplementary Figure S2A). Using ExoCounter [29,30,31], a high-sensitivity Blu-ray-based EV quantification platform (Figure 5A), NHRI#20D5 captured over twice as many CD98+ EVs as the Abcam antibody and threefold more than the Novus antibody (Supplementary Figure S2B). These results validated NHRI#20D5 as a high-affinity reagent for detecting EV-surface CD98.

### 3.3. CD98+ Extracellular Vesicles Visualized at Single-Vesicle Resolution

**Figure 4:**
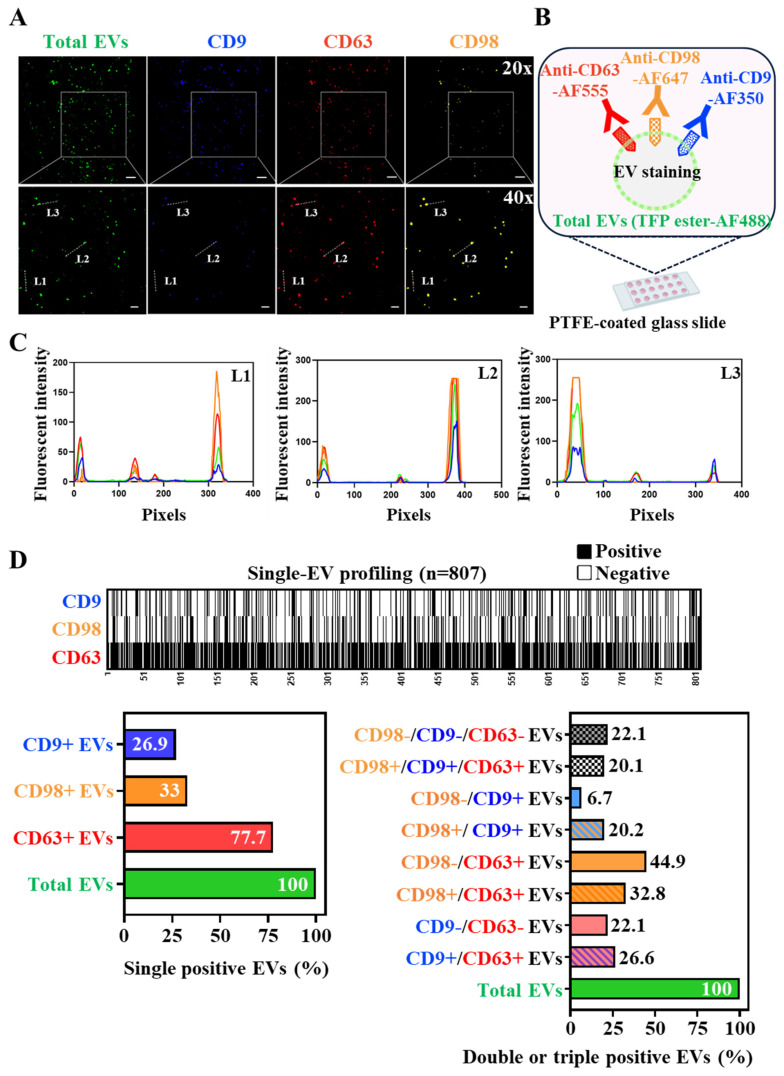
CD98 co-localizes with classical exosome markers CD63 and CD9 in HepG2-derived EVs. (**A**) Representative images of CD63, CD9, and CD98 surface staining, along with total EV staining by TFP ester–AF488, in HepG2-derived EVs using single-EV imaging at 20× (**upper** panel) and 40× (**lower** panel). Scale bars = 50 µm and 20 µm, respectively. (**B**) Illustration of the multiplex fluorescence imaging principle of the single-EV imaging technique. (**C**) Line scans (L1–L3) from (**A**) were analyzed by ImageJ 1.54p to display relative fluorescence intensity (RFU) of TFP ester, CD63, CD9, and CD98 per pixel. (**D**) Single-EV profiling of 20× images (*n* = 3) quantifying the proportion of positive EVs for each marker (CD63, CD9, and CD98) or marker combination relay on TFP ester–AF488-positive signal (total EVs).

We next visualized CD98+ EVs using single-EV optical imaging [32,33]. Enriched HepG2-derived EVs were co-stained for CD63, CD9, and CD98. Enriched HepG2-derived EVs were co-stained for CD63, CD9, and CD98. To ensure the accuracy of single-EV quantification, we confirmed via nanoparticle tracking analysis (NTA) that the enriched EVs from HepG2-conditioned media did not form aggregates. Furthermore, total EV staining was performed using a confocal microscope with stimulated emission depletion (STED) to achieve super-resolution imaging (20–50 nm) (Supplementary Figure S3A). Image analysis revealed that 33.0% of EVs were CD98+, 26.9% were CD9+, and 77.7% were CD63+ (Figure 4A,B). Line scan analyses (L1, L2, and L3) of enlarged images (40×) (Figure 4C) confirmed the co-localization of CD98 with canonical EV markers. Quantitative single-EV profiling (*n* = 807 from three 20× images) (Figure 4D) demonstrated that most CD98+ EVs were either CD98+/CD63+ or CD98+/CD9+ double-positive. Notably, distinct populations of CD63+ and CD9+ EVs were CD98-negative (44.9% and 6.7% of total EVs, respectively), whereas 22.1% represented a triple-negative subpopulation (CD63-/CD9-/CD98-). These findings indicate that CD98+ EVs mark a unique subpopulation of classical EVs.

### 3.4. CD98+ EVs Are Elevated in Plasma of Patients with Early HCC

**Figure 5:**
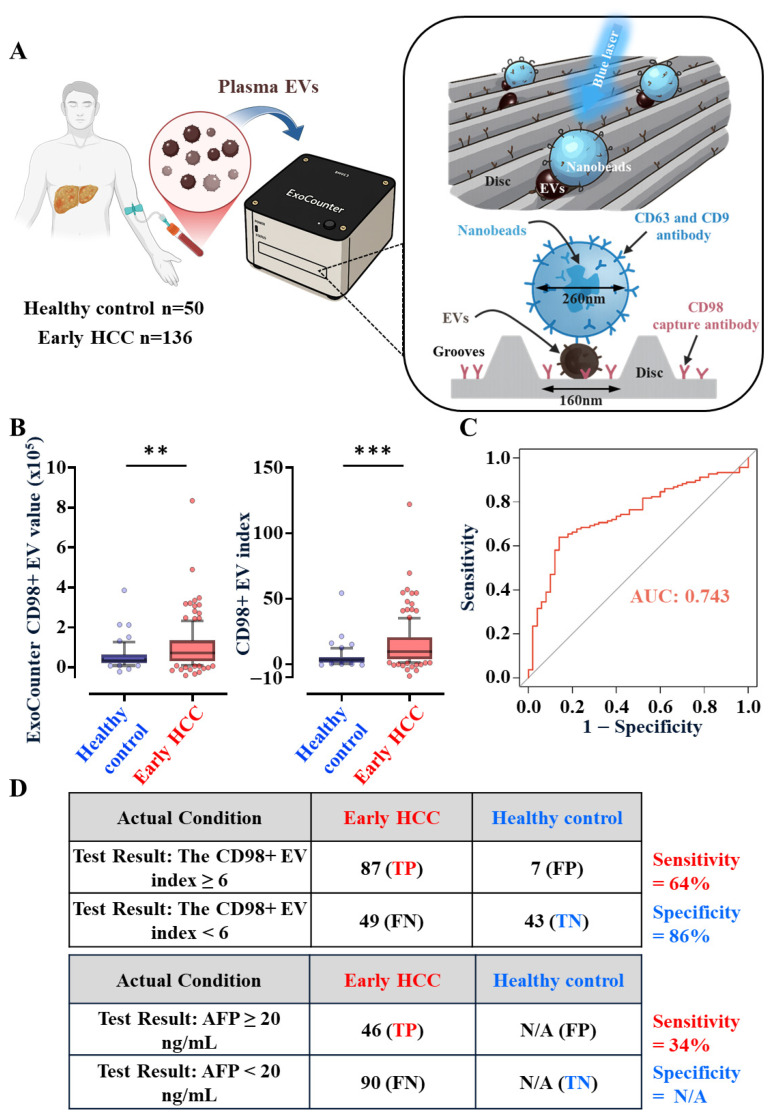
Identification of plasma CD98+ EVs as a novel diagnostic biomarker for early-stage HCC. (**A**) Schematic illustrating plasma CD98+ EV quantification using ExoCounter technology. (**B**) Original CD98+ EV counts from patient plasma (left) and normalized CD98+ EV index (right) are shown as dot plots. ** = *p* < 0.01, *** = *p* < 0.001 (Student’s *t* test). (**C**) ROC curve showing diagnostic performance of the CD98+ EV index. (**D**) Sensitivity and specificity of the CD98+ EV index (cutoff = 6) were calculated from true/false positive and negative rates in early HCC. AFP (cutoff = 20 ng/mL) was compared as a reference, though AFP data were unavailable for healthy controls. TP = true positive; TN = true negative; FP = false positive; FN = false negative.

We applied ExoCounter analysis (Figure 5A) to plasma EVs from healthy individuals (*n* = 50) and patients with early-stage non-viral HCC (*n* = 136; Stage I/II) (Table 1). The HCC cohort exhibited typical demographic and lifestyle risk factors, including older age, male predominance, and higher smoking/alcohol exposure. Importantly, indicators of liver cirrhosis and fibrosis (AAR, APRI) and lipid profiles (CHO, TG) suggested overall preserved liver function and minimal hepatic steatosis, validating this cohort for early-stage analysis.

**Figure 6:**
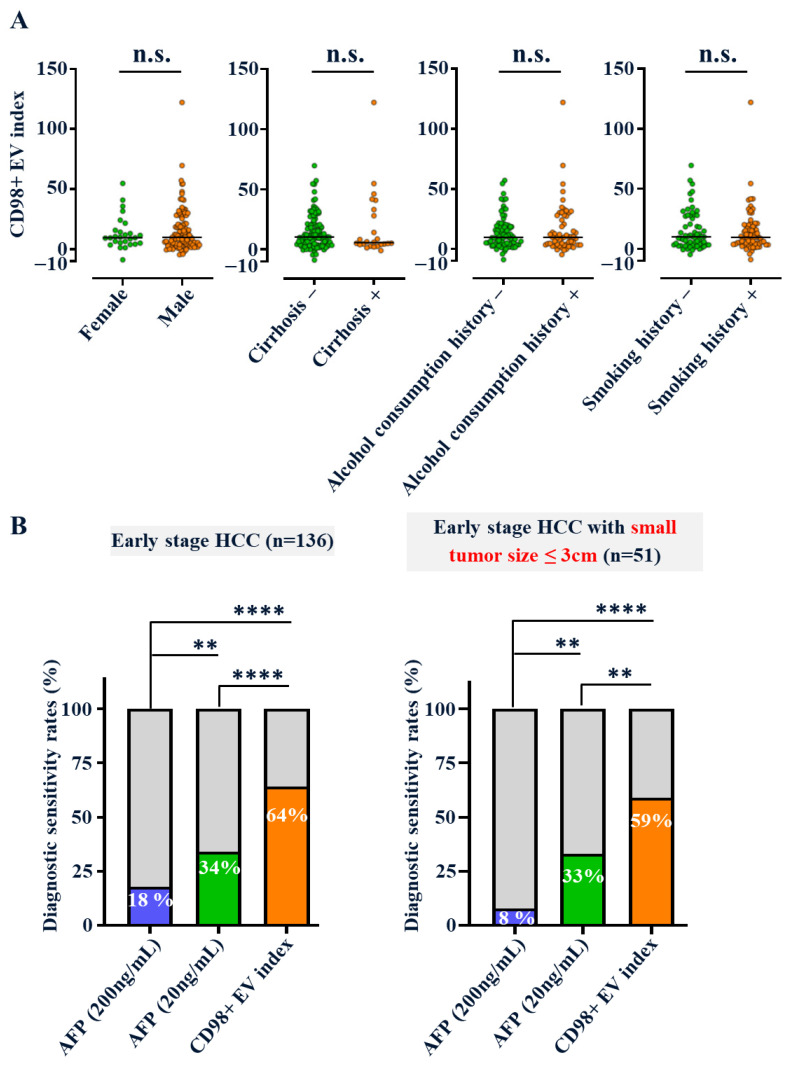
The CD98+ EV index is an independent and early detection biomarker. (**A**) In the non-viral early HCC cohort, CD98+ EV index values were compared across subgroups with different risk factors, including sex, smoking, alcohol consumption, and cirrhosis. Data are shown as dot plots. No significant differences (n.s.) were observed between groups (Student’s *t* test). (**B**) Bar graphs show diagnostic sensitivity (%) for blood AFP (200 ng/mL and 20 ng/mL), and CD98+ EV index in early HCC (stage I/II, *n* = 136) and small tumors (≤3 cm, *n* = 51). ** = *p* < 0.01, **** = *p* < 0.0001 (Z-score test for two proportions).

All plasma samples underwent standardized preparation, including high-speed centrifugation, size-exclusion chromatography (SEC), and NTA, to determine EV concentration and size (Supplementary Figure S3B). The observed EV size (145–165 nm) was within the characteristic exosome range. ExoCounter quantification revealed significantly elevated CD98+ EV counts in early HCC plasma compared with healthy controls (*p* = 0.0061) (Figure 5B, left). After normalization by total EV concentration, the resulting “CD98+ EV Index” further enhanced group separation (*p* = 0.0001) (Figure 5B, right). No association was observed between the CD98+ EV Index and smoking, alcohol use, cirrhosis, age, sex, AFP, tumor stage, or size (Figure 6A, Table 2), confirming its etiology-independent diagnostic potential.

The ROC curve yielded an AUC of 0.743, with 64% sensitivity and 86% specificity for early HCC detection (Figure 5C,D). Notably, for tumors ≤3 cm, the CD98+ EV Index maintained 59% sensitivity, significantly outperforming AFP (8% or 33% at cutoffs of 200 or 20 ng/mL, respectively; Figure 6B). Furthermore, a logistic regression model with 5-fold stratified cross-validation was used to evaluate predictive performance (Supplementary Figure S4B). Together, these results demonstrate that the CD98+ EV Index provides a robust, noninvasive, and highly sensitive biomarker for detecting early-stage non-viral HCC, surpassing the performance of conventional AFP testing.

## 4. Discussion

In this study, we developed CD98-positive extracellular vesicles (EVs) as a novel blood-based biomarker for hepatocellular carcinoma (HCC) using ExoCounter technology. Through single-EV imaging, we confirmed that in HepG2 cells, surface CD63 and CD9 are the major tetraspanins associated with HCC-secreted EVs, while CD98 is present on approximately 31.2% of these vesicles. The co-localization of CD98 with CD63 and CD9 on individual EVs was clearly demonstrated. To our knowledge, this is the first study to quantify CD98+ EVs in HCC and evaluate their utility as an early diagnostic biomarker, although the oncogenic role of CD98 is well-established across multiple cancer types. A limitation of our study is the lack of data regarding whether the CD98+ EV Index is also elevated in other malignancies, such as lung, colon, or pancreatic cancers. Further studies are warranted to assess the specificity and diagnostic value of CD98+ EVs across diverse tumor types.

Existing literature suggests that CD98+ EVs may contribute to tumorigenesis through exosome-mediated signaling. For instance, CD98 forms complexes with integrin proteins in healthy dermal fibroblast-derived EVs to promote wound healing [34]. Conversely, tumor-derived CD98+ EVs from extranodal natural killer/T-cell lymphoma have been shown to enhance proliferation, invasion, and drug resistance [35]. Moreover, CD147, a known surface-interacting partner of CD98 [36,37,38], drives proliferation, migration, and invasion in rhabdomyosarcoma [39]. Plasma CD147+ EVs have also been identified as potential diagnostic and prognostic biomarkers for gastric and colorectal cancers [40,41]. Given the established oncogenic functions of CD98 in regulating integrin/FAK/PI3K and mTOR signaling, EV-associated CD98 may similarly participate in these pathways upon delivery to recipient cells. Although this study focuses on the diagnostic utility of CD98+ EVs, future research should investigate their functional roles in HCC progression.

The unfavorable prognosis of HCC is strongly associated with large tumor size and advanced disease stage. Notably, CD98 mRNA expression is independent of survival, tumor size, and stage, supporting its potential role in early HCC detection (Figure 1B,C). The CD98+ EV Index demonstrated excellent diagnostic performance in distinguishing early-stage HCC (tumor size ≤ 3 cm) (Figure 6B). Currently, ultrasound (US) and alpha-fetoprotein (AFP) testing are the standard screening tools for HCC, but both are suboptimal for detecting small, early-stage tumors. In our clinical cohort, the CD98+ EV Index (64%) achieved diagnostic sensitivities approximately two to three times higher than those of AFP (18–34%) for early HCC (Figure 6B). This finding aligns with the poor sensitivity of AFP (13–30%) for early HCC (BCLC 0 & A) observed in the CLCA dataset, where PIVKA-II (DCP) performed better (38.5%) (Supplementary Figure S1). Inspired by the GALAD [42,43] and ASAP [44,45] models, combining AFP and PIVKA-II can improve diagnostic sensitivity [10,14], and future studies should explore whether combining CD98+ EV Index with AFP and PIVKA-II could further enhance both sensitivity and specificity. Such a multimodal approach may also help address the potential confounding effect of elevated CD98+ EVs in other CD98-abundant cancers.

Surface proteins on EVs have attracted considerable attention as minimally invasive cancer biomarkers. The ExoCounter platform provides quantitative and qualitative assessment of EV surface proteins with several advantages, including size-based EV selection through nanogrooves in Blu-ray discs (Figure 3C), multiplexing capacity for up to 16 samples per run, and no need for EV isolation, enabling analysis from small sample volumes. These features make ExoCounter a superior choice compared with fluorescence nanoparticle tracking analysis or nano-flow cytometry. Continued development of multiplex EV profiling technologies—such as immuno-droplet digital polymerase chain reaction (iddPCR) [46], single-EV analysis (SEA) [47], multiplexed analysis of EVs (MASEV) [48], and impedance profiling of EVs (iPEX) [49]—will further expand our ability to profile EV subpopulations like CD98+ EVs.

In conclusion, our study demonstrated elevated CD98 expression in HCC tumors and the presence of CD98+ EVs secreted by HCC cell lines. Using the ExoCounter system and our custom anti-CD98 antibody, we identified significantly higher levels of CD98+ EVs in HCC patients than in healthy controls. The CD98+ EV Index exhibited a reliable AUC and excellent sensitivity for detecting small, early-stage HCC (Figure 6B). Together, these findings highlight CD98+ EVs as a promising blood-based biomarker for early HCC detection, potentially improving survival outcomes in the growing population at risk of non-viral HCC. Most importantly, comprehensive validation in larger, multi-stage patient cohorts will be essential to facilitate its translation into clinical practice.

## 5. Conclusions

In conclusion, this study identifies CD98-positive extracellular vesicles as a promising noninvasive biomarker for the detection of hepatocellular carcinoma, particularly in nonviral cases. CD98 levels were significantly elevated in patients with early-stage disease and demonstrated superior performance compared with conventional markers, especially in detecting small tumors. These findings highlight the potential of CD98 as a robust diagnostic indicator across different disease stages and etiologies.

The integration of CD98-based extracellular vesicle detection with a sensitive analytical platform provides a clinically applicable approach for improving early diagnosis. This strategy may facilitate more accurate risk stratification and support timely clinical decision-making. Further validation in larger and independent cohorts is warranted to confirm its clinical utility and to explore its role in routine screening and precision oncology.

## Figures and Tables

**Table 1:** Clinical characteristics of early HCC patients (Stage I/II, non-HBV/HCV HCC) and healthy controls.

	Healthy Control Median (IQR)	Non-viral HCC Patients Median (IQR)
	*n* = 50	*n* = 136
Age	38.5 (11.25)	66 (4.75)
CD98+ EV index	3.548 (3.821)	9.761 (16.405)
Platelet (10^3^/uL)	225.5 (57.8)	205 (91.5)
CHO (mg/dL)	192 (34.3)	163 (47.3)
TG (mg/dL)	75 (76)	98 (61.5)
AST (U/L)	20 (3)	30.5 (18.75)
ALT (U/L)	19 (5.5)	29 (24.75)
AAR	1 (0.355)	1 (0.5275)
APRI	0.22 (0.0725)	0.38 (0.28)
Biological sex (Male/Female)	n = 28 (56.0%)/n = 22 (44.0%)	n = 109 (80.1%)/n = 27 (19.9%)
Smoking history (Yes/No)	n = 17 (34.0%)/n = 33 (66.0%)	n = 65 (47.8%)/n = 71 (52.2%)
Alcohol consumption history (Yes/No)	n = 10 (20.0%)/n = 40 (80.0%)	n = 60 (44.1%)/n = 76 (55.9%)

Abbreviations: IQR, Interquartile range; CHO, Cholesterol; TG, Triglycerides; AST, Aspartate aminotransferase; ALT, Alanine aminotransferase; AAR, Aspartate-to-alanine aminotransferase ratio; APRI, AST-to-Platelet Ratio Index.

**Table 2:** Correlation between plasma CD98+ EV index and clinical variables in early HCC.

	**Higher CD98 Index** **Mean ± std.**	**Lower CD98 Index** **Mean ± std.**	**Number (High/Low)**		***p* Value**
Age (years)	66.21 ± 10.61	66.60 ± 9.72	68/68		0.820
Platelet (10^3^/uL)	209.98 ± 80.08	216.92 ± 69.29	58/63		0.613
CHO (mg/dL)	162.17 ± 34.25	171.66 ± 47.84	29/29		0.390
TG (mg/dL)	110.80 ± 50.31	119.05 ± 86.70	25/20		0.708
AFP (ng/ml)	119,367.51 ± 969,973.83	575.37 ± 3150.90	68/68		0.316
AST (U/L)	32.54 ± 16.56	38.56 ± 27.62	68/68		0.126
ALT (U/L)	33.90 ± 21.05	41.99 ± 50.06	68/68		0.223
Tumor size(cm)	5.36 ± 3.95	5.25 ± 3.72	68/68		0.869
Tumor number	1.09 ± 0.29	1.31 ± 1.08	68/68		0.108
**Categorical variable**	**Higher CD98 Index (***n***)**	**Lower CD98 index (***n***)**	**χ^2^ (df)**		***p* Value**
Biological sex	68	68	0.046	(1)	0.830
Smoking history	68	68	0.030	(1)	0.864
Alcohol consumption history	68	68	0.000	(1)	1.000
Cirrhosis	68	68	3.238	(1)	0.072
Tumor grade	68	68	0.825	(3)	0.843
Tumor stage	68	68	0.032	(1)	0.858
Vascular invasion	68	68	1.826	(3)	0.609

Abbreviations: CHO, Cholesterol; TG, Triglycerides; AST, Aspartate aminotransferase; ALT, Alanine aminotransferase.

## Data Availability

The original contributions presented in this study are included in the article/Supplementary Material. Further inquiries can be directed to the corresponding author.

## Supplementary Materials

The following supporting information can be downloaded at: https://www.mdpi.com/article/10.3390/cancers18071086/s1, Figure S1: Limited diagnostic performance of AFP and PIVKA-II for early HCC detection. From the CLCA cohort [21] via cBioPortal [24], diagnostic sensitivity (%) of blood AFP (cutoffs: 200 ng/mL and 20 ng/mL) and PIVKA-II (cutoff: 40 mAU/mL) for early (BCLC 0 & A, n = 28) versus late HCC (BCLC B, n = 81; BCLC C, n = 130) were displayed as bar graphs; Figure S2: Comparison of three anti-CD98 antibodies for detecting extracellular CD98 on cells and EVs. (A) Surface CD98 on HepG2 cells quantified using three antibodies (Abcam ab307587, Novus NBP2-36491SS, NHRI#20D5) by flow cytometry. NHRI#20D5 specificity was validated via neutralization with recombinant CD98 extra-cellular domain protein. (B) CD98+ EVs isolated from HepG2-conditioned medium were measured using anti-CD63/CD9 nanobeads and anti-CD98 antibodies (Abcam, Novus, NHRI#20D5) with ExoCounter analysis; Figure S3: Determination of EV concentration and size of HepG2 cell culture media and plasma. (A) EV concentration and size in HepG2 cell culture media by nanoparticle tracking analysis (NTA) in the left bar plot and super-resolution STED imaging of HepG2 extracellular vesicles in the right image. Scale bar is 500 nm. (B) EV concentra-tion and size in plasma samples from early HCC (n = 136) and healthy controls (n = 50) were plotted. n.s. = not significant; ** = *p* < 0.01 (Student’s *t* test); Figure S4: Internal validation by sub-grouping according to age or gender, and by a logistic regression model with 5-fold stratified cross-validation. (A) CD98+ EV index from patient plasma with similar age range or gender sub-groups are shown as dot plots. * = *p* < 0.05, ** = *p* < 0.01 (Student’s *t* test). (B) Receiver operating characteristic (ROC) curves with 5-fold stratified cross-validation were generated. The average area under the ROC curve (AUC) is 0.73 with a standard deviation of 0.1; Figure S5: Full-length unprocessed blots for Figure 3; Figure S6: Band intensity of Western blots for Figure 3.
