# Supplementary material for: Blu-Ray-Based Quantification of CD98+ Extracellular Vesicles for Early Detection of Hepatocellular Carcinoma
Source: Cancers (Basel). 2026 Mar 26;18(7):1086. doi: 10.3390/cancers18071086 (PMC13072036; doi:10.3390/cancers18071086)
Supplement: Supplementary file 1 [file cancers-18-01086-s001.zip › cancers-4125149-supplementary.pdf]

cancers

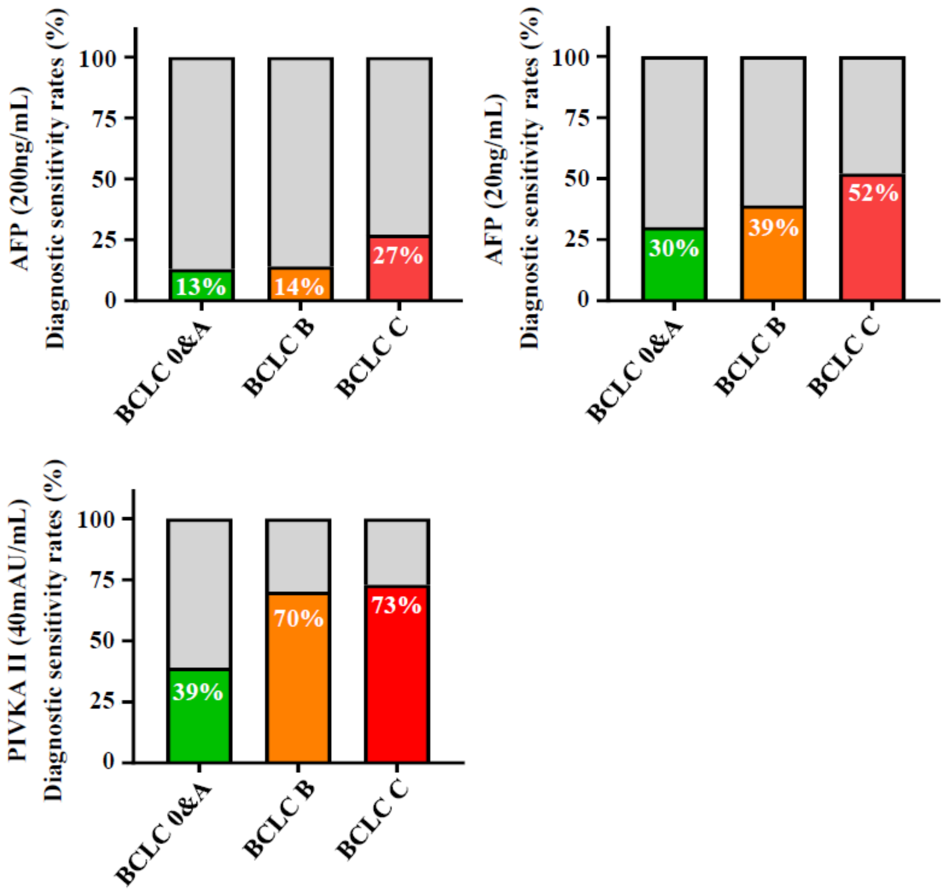


**Supplementary Figure S1. Limited diagnostic performance of AFP and PIVKA-II for early HCC detection.**
From the CLCA cohort [21] via cBioPortal [24], diagnostic sensitivity (%) of blood AFP (cutoffs: 200 ng/mL and 20 ng/mL) and PIVKA-II (cutoff: 40 mAU/mL) for early (BCLC 0 & A, n = 28) versus late HCC (BCLC B, n = 81; BCLC C, n = 130) were displayed as bar graphs.

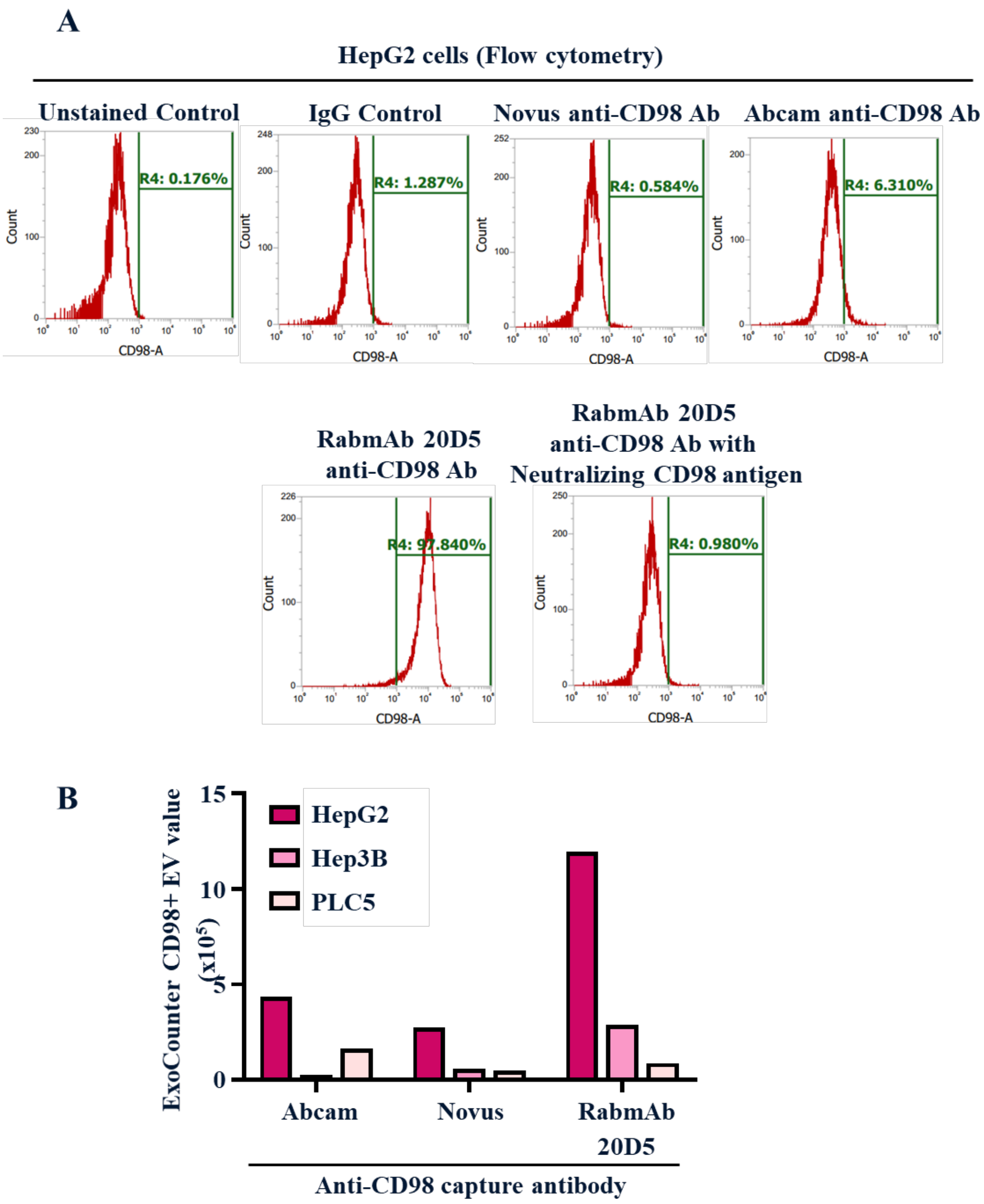


**Supplementary Figure S2. Comparison of three anti-CD98 antibodies for detecting extracellular CD98 on cells and EVs. (A)** Surface CD98 on HepG2 cells quantified using three antibodies (Abcam ab307587, Novus NBP2-36491SS, NHRI#20D5) by flow cytometry. NHRI#20D5 specificity was validated via neutralization with recombinant CD98 extracellular domain protein. **(B)** CD98+ EVs isolated from HepG2-conditioned medium were measured using anti-CD63/CD9 nanobeads and anti-CD98 antibodies (Abcam, Novus, NHRI#20D5) with ExoCounter analysis.

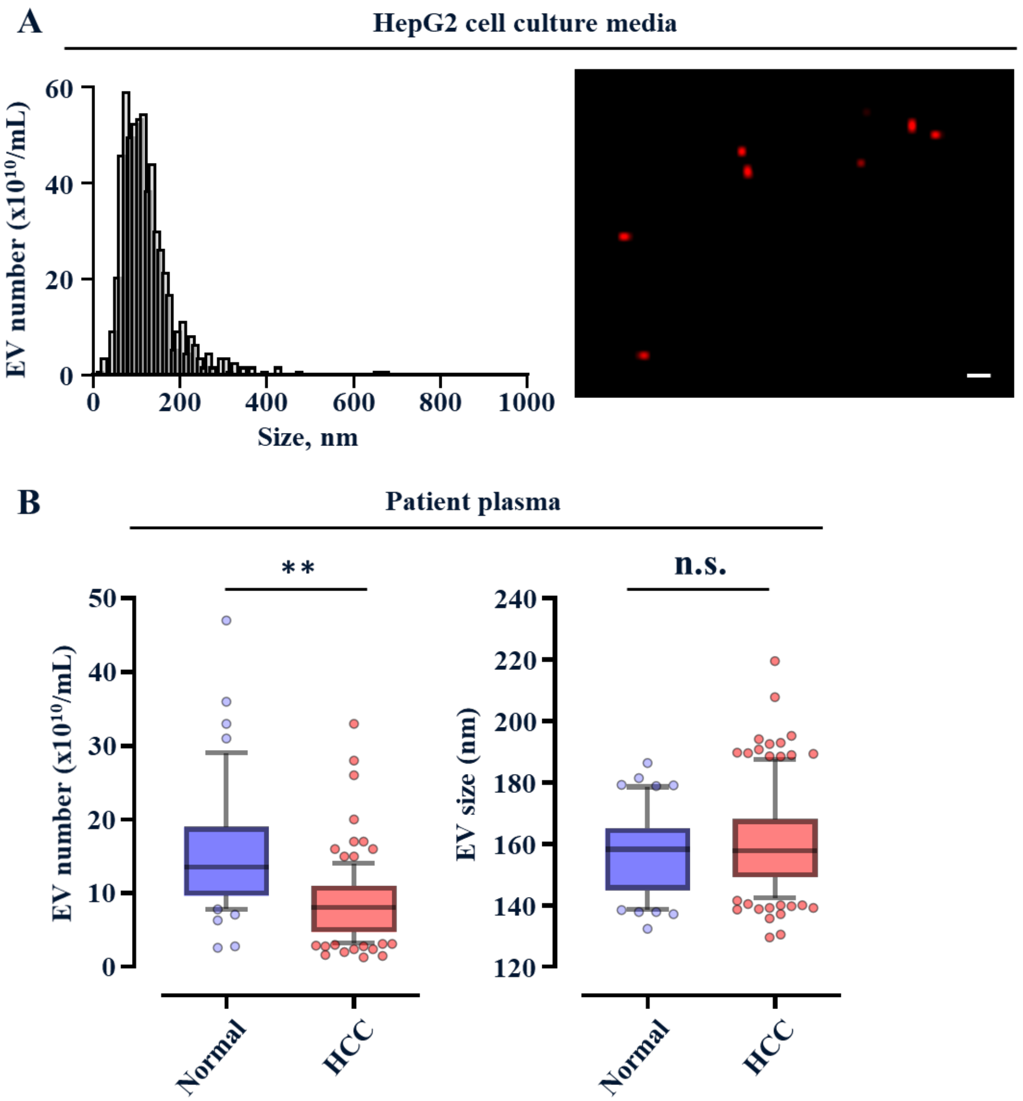


**Supplementary Figure S3. Determination of EV concentration and size of HepG2 cell culture media and plasma.**
**(A)** EV concentration and size in HepG2 cell culture media by nanoparticle tracking analysis (NTA) in the left bar plot and super-resolution STED imaging of HepG2 extracellular vesicles in the right image. Scale bar is 500nm. **(B)** EV concentration and size in plasma samples from early HCC (n = 136) and healthy controls (n = 50) were plotted. n.s. = not significant; ** = $p < 0.01$ (Student's t test).

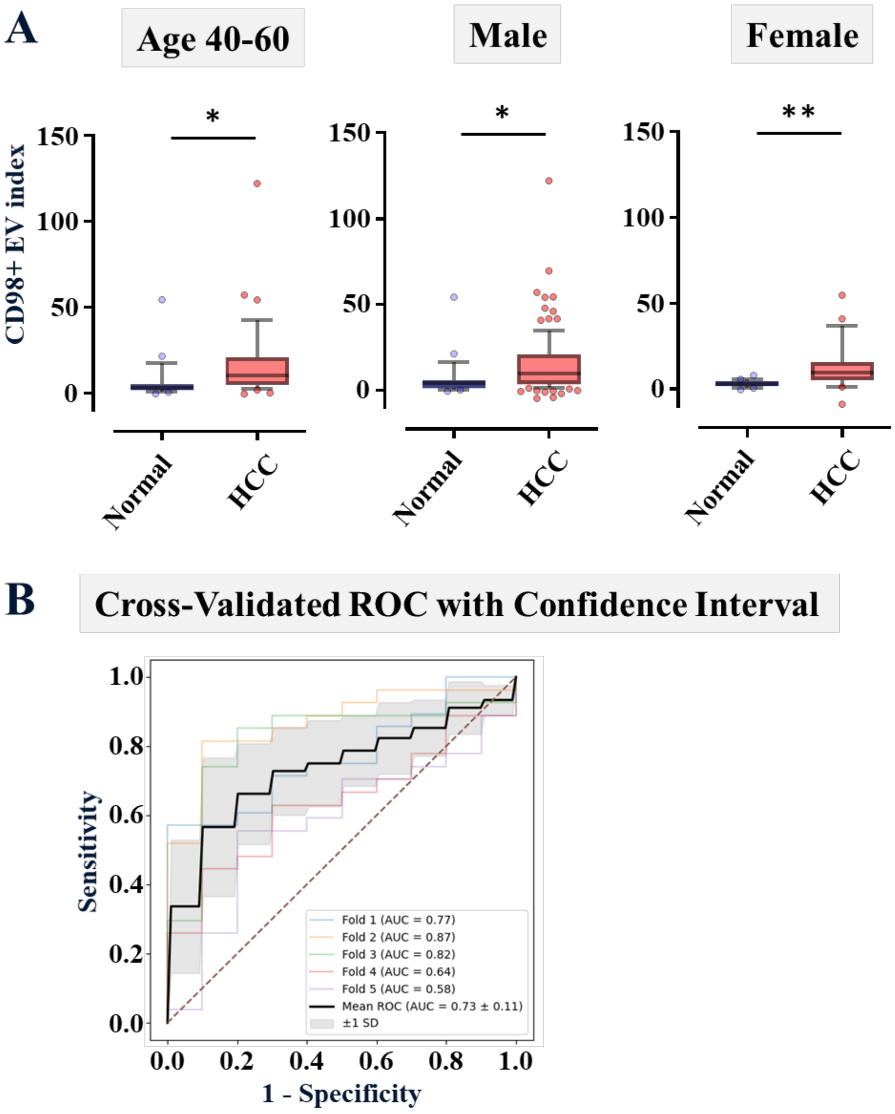


**Supplementary Figure S4. Internal validation by sub-grouping according to age or gender, and by a logistic regression model with 5-fold stratified cross-validation.**

**(A)** CD98+ EV index from patient plasma with similar age range or gender sub-groups are shown as dot plots. * = $p < 0.05$, ** = $p < 0.01$ (Student's t test). **(B)** Receiver operating characteristic (ROC) curves with 5-fold stratified cross-validation were generated. The average area under the ROC curve (AUC) is 0.73 with a standard deviation of 0.1.

.

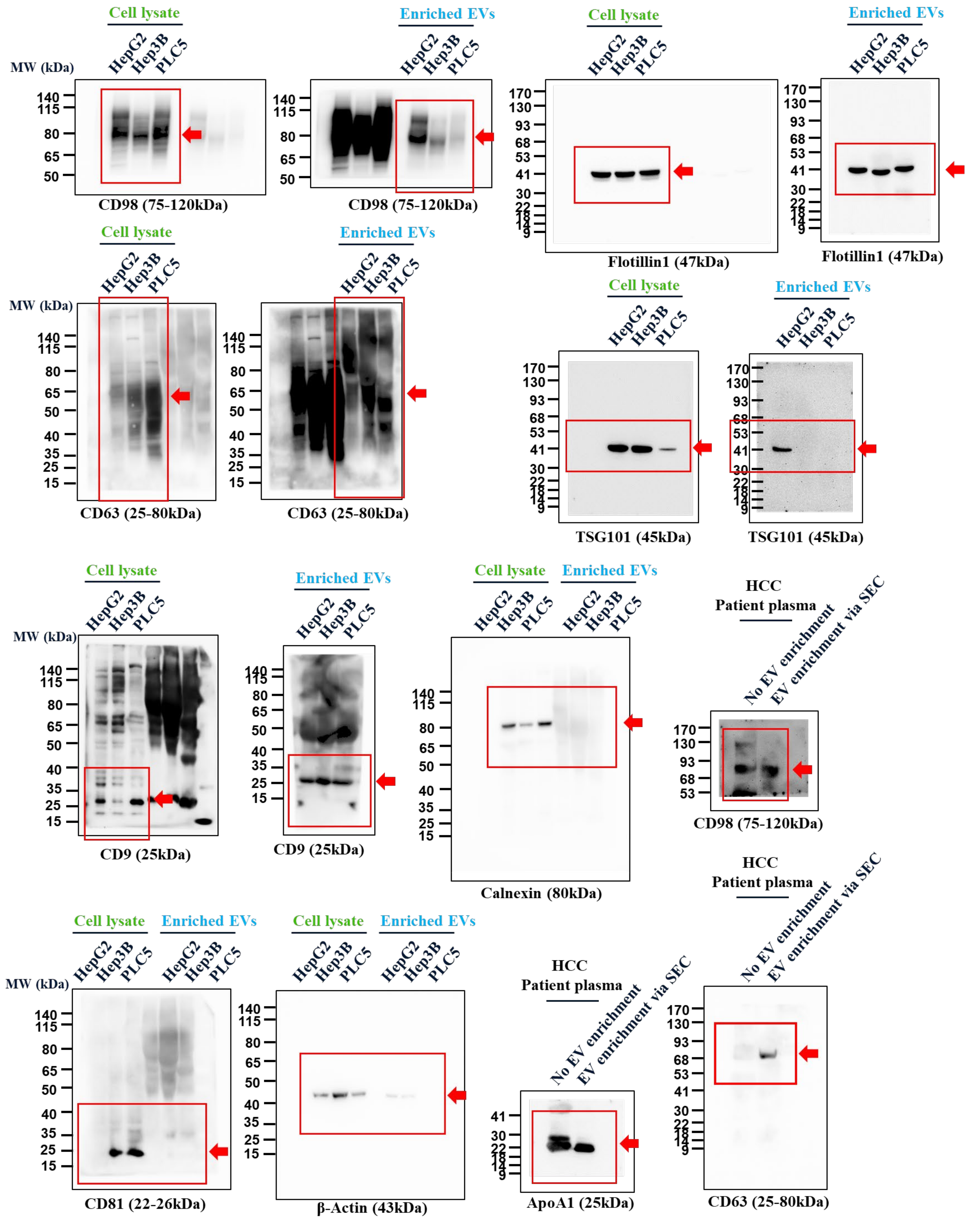


**Supplementary Figure S5. Full-length unprocessed blots for Figure 3.**

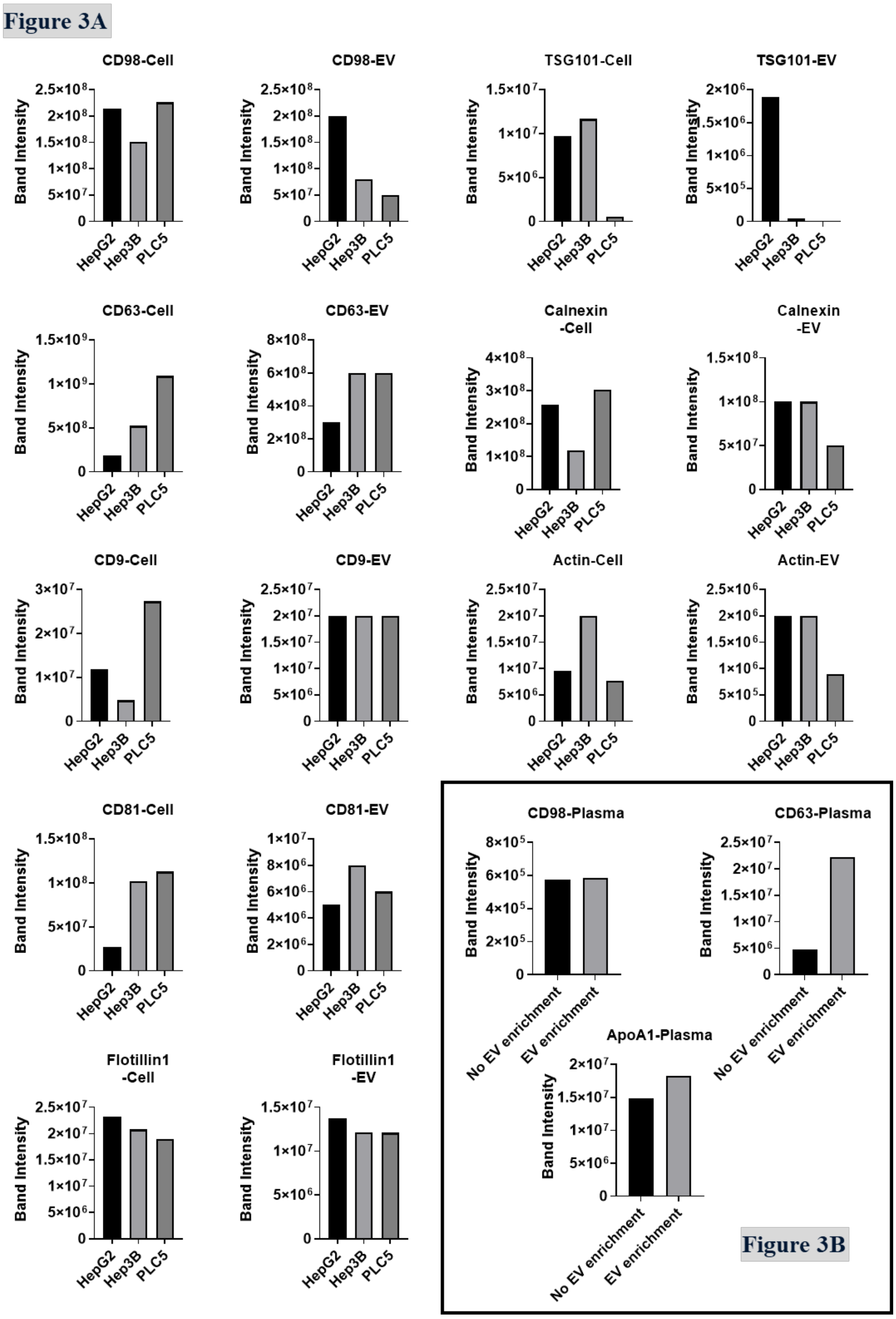


**Supplementary Figure S6. Band intensity of Western blots for Figure 3.**
